# Prescriptions of antibiotics in out-of-hours primary care setting in Reykjavik capital area

**DOI:** 10.1080/02813432.2020.1794159

**Published:** 2020-07-16

**Authors:** Holmfridur Asta Palsdottir, Jon Steinar Jonsson, Emil L. Sigurdsson

**Affiliations:** a Primary Health Care of the Capital Area, Reykjavik, Iceland; b Department of Family Medicine, University of Iceland, Reykjavik, Iceland; c Development Centre for the Primary Care, Reykjavik, Iceland

**Keywords:** Out-of-hours service, primary care, antibiotics, Iceland, drug prescriptions, general practice

## Abstract

**Objective:**

To describe antibiotic prescriptions in out-of-hour (OOH) service in primary care setting in Iceland and to study the indications for prescriptions.

**Design:**

A population based retrospective study, using electronic data from the OOH registration system.

**Setting:**

OOH primary care setting in Reykjavik capital area in Iceland.

**Subjects:**

All patients that received a prescription for oral antibiotic drug at an OOH service in Reykjavik capital area over a one-year period.

**Main outcome measures:**

Number of oral antibiotic prescriptions and diagnosis connected to the prescriptions according to age and sex.

**Results:**

There were 75,582 contacts with the OOH primary care of which 25,059 contacts resulted in prescription of an oral antibiotic (33%). The most common antibiotic prescribed in total, and for the diagnosis studied, was amoxicillin with clavulanic acid. It was most often prescribed for acute otitis media. Of those diagnosed with otitis media 50% were treated with amoxicillin with clavulanic acid and 40% of those diagnosed with pneumonia received that treatment. The second most prescribed antibiotic was amoxicillin. Most often it was prescribed for sinusitis, in 47% of cases with that diagnosis.

**Conclusion:**

Antibiotics are often prescribed in OOH primary care in Iceland and a substantial number of the patients diagnosed in OOH primary care with acute otitis media or pneumonia are prescribed broad-spectrum antibiotics.

**Key points**
- Antibiotic prescription rate is high and broad-spectrum drugs often prescribed in OOH primary care service in Iceland.
- The results should encourage general practitioners in Iceland to review antibiotic prescriptions in OOH service.

## Introduction

Antibiotic resistance has been an increasing problem in the whole world during the last decades [1]. Health organisations and some of the world political leaders have expressed their concerns and consider this problem as one of the biggest threat of our time [2]. Increased prescriptions of antibiotics and increased exposure to antibiotics is considered to play a major role in this increasing antibiotic resistance [3]. In Iceland the major part of antibiotic prescriptions goes through primary care [4] and studies confirm the same for its neighbour countries [5–8]. Furthermore, most antibiotic prescriptions goes through outpatient clinics (88%), and General Practitioner´s (GP´s) in Iceland prescribe 58% of all antibiotics prescribed in the country [4]. Icelanders have the highest rate of antibiotic prescriptions among the Nordic countries, 22 Defined Daily Dose (DDD)/1000 inhabitants/day in 2015, and out of those 19,4 DDD/1000 inhabitants/day received prescription through outpatient clinics [4]. In a European study project group on outpatient antibiotic use in Europe from 1997–2009, the median outpatient antibiotic use was 19.0 DDD/1000 inhabitants/day among 33 European countries, where Penicillins were the most frequently prescribed antibiotics in all 33 countries [9]. Iceland was among the countries with the lowest prescription rate of quinolones (3%) and the highest prescription rate of tetracyclines (26%) among the total outpatient use [9]. Compared to other European countries, antibacterial resistance in Iceland is low for E.Coli non-susceptible for fluoroquinolones, and high for S.pneumoniae non-susceptible for penicillin, compared to other European countries [10].

Increased antibiotic prescriptions in out-of-hour (OOH) services in general practice has been observed over the last years and decades in several European countries [9,11–13]. Claims have been made that there are more prescriptions of antibiotics in OOH service than daytime service [8,11,13–15]. Some studies have revealed that more broad-spectrum antibiotics are prescribed in OOH service than daytime service [8,11,13,15]. One study from England showed that despite a reduction in the number of patient contacts with the OOH service, antibiotic prescriptions from OOH service rose during the study period [5]. A matching increase was not seen for daytime antibiotic prescriptions [5]. A study from The Netherlands revealed that although there were higher rates of antibiotic prescriptions in OOH service, the overprescribing was comparable, or even lower than for daytime practice [11].

Acute Respiratory Tract infections (ARTIs) are common reasons for consulting the OOH services and are considered to account for up to 60% of all OOH consultations [6,7,12,14,16]. Clinical guidelines for antibiotic prescription for ARTIs are different between countries. In Denmark, Norway and Sweden, where antibiotic resistance is low [7,12,14], penicillin V is recommended as a first-line antibiotic to treat ARTIs, if antibiotic is deemed necessary [7,12,14]. In the Netherlands, penicillin V is only recommended as a first-line antibiotic in acute tonsillitis, but in other forms of ARTIs where antibiotics are required, amoxicillin or doxycycline are recommended as a first-line antibiotic [11]. In Iceland, there have only been published clinical guidelines on antibiotic treatment for otitis media (OM), where the first-line antibiotic is amoxicillin [17].

To promote rational antibiotic prescriptions in Iceland, especially for OOH service, we firstly have to analyse and describe the antibiotic prescriptions in OOH service and see how they are compared to clinical guidelines from other countries and for OM in Iceland.

The aim of this study is to describe antibiotic prescriptions in OOH primary care in Reykjavik capital area over a one-year period and analyse them by patient age, gender and diagnosis.

## Material and methods

All individuals who contacted the OOH primary care service in Reykjavik capital area from January 1^st^ 2014 to December 31^st^ 2014, and were prescribed an oral antibiotic (J01) formed the study group. The study period was chosen to ensure a coherent winter season with seasonal peaks, such as influenza.

Total population in Iceland on January 1^st^ 2015 was 329,100 [18]. Reykjavík capital area has the highest population in the country, 201,766 inhabitants on January 1^st^ 2015 (61.3%) [18]. OOH service for primary care, is provided in one place for the area, from 17:00-23:30 on weekdays and from 8:00–23:30 on weekends and holidays. From 23:30 to 8:00, there is a telephone consultation with a nurse but no doctor is on call and people are referred to the Emergency Department at the University Hospital in Reykjavík, if they need urgent care. Consultations are either office visits or home visits. There are no telephone consultations with a GP at the OOH primary care service in Reykjavík capital area.

This is a population based retrospective study. All the data was taken from the OOH electronic medical registration system. OOH registration system is fully electronical and each contact is registred through the civil registration number, assigned to every citizen in Iceland. Information was gathered about the patient age, gender and type of consultation. Furthermore, we could provide data from the electronic medical system, for the ATC coding system (Anatomic Therapeutic Chemical Classification System) for oral antibiotics (J01) and connect them with the disease diagnosis by the ICD-10 code (WHO International Classification of Diseases, version 2010). Antibiotic prescriptions for the following diagnosis, in alphabetical order, was specifically studied; bronchitis (ICD10 code J20 and J40), cystitis (ICD10 code N30-N30.9, N34-N34.1, N36.9, N39.0-N39.4), otitis media (ICD10 code H65-H65.9, H66.0-H66.9, H68, H70.9, H72, H73.0), pneumonia (ICD10 code J12-J18) and sinusitis (ICD10 code J32-J32.1, J01-J01.0). Age was categorised into following age groups; 0–4 years (age group 1), 5–17 years (age group 2), 18–60 years (age group 3) and >60 years (age group 4).

The data was collected in Excel 2010 and SPSS (version 24) was used to perform the statistical analysis. We used descriptive statistic to describe the data.

The data was not personally identifiable. Authorization for this study was given by The National Bioethics Committee in Iceland, in December 2015 (VSN-15-162).

## Results

During the study period, there were 75,582 contacts with the OOH primary care service in Reykjavik capital area. Office clinical consultations were 72,360 (95.7%) and home visits 3222 (4.3%). Of those, 25,059 contacts resulted in a prescription of an oral antibiotic (33.1%). The number of individuals who were prescribed an oral antibiotic was 19,641, therefore, 5418 individuals (7.2%) got more than one prescription of an oral antibiotic during the study period. Antibiotic prescriptions through office consultation were 24,286 (96.9%), and 766 (3.1%) through home visits. Seven antibiotic prescriptions were given after a telephone consultation through a nurse.

Mean age of the participants was 32.4 year (0.0–99.0; SD 21.6). Males were 10,346 (41.3%) and females 14,712 (58.7%). In all age groups, females were often prescribed an antibiotic, except for the youngest age group, where males were 52.7% of those prescribed an antibiotic.

Of the diagnosis we selected in this study, there were in total 12.850 prescriptions of 17 different oral antibiotics (Table 1), 51.3% of all antibiotic prescriptions in OOH service and 17% of all contacts in OOH service. An antibiotic was most often prescribed for sinusitis, 27.4% of total antibiotic prescriptions, and secondly for bronchitis, 24.9% of total antibiotic prescriptions in this study.

**Table 1.** Number of all antibiotics prescribed per diagnosis (% of antibiotic per diagnosis) *.

ATC code	Drug	Otitis Media	Cystitis	Sinusitis	Bronchitis	Pneumonia	Total *n* (% of total)
J01CR02	Amoxicillin and clavulanic acid	1361 (36.7)	13 (0.4)	1115 (30.0)	707 (19.1)	512 (13.8)	3708 (28.9)
J01CA04	Amoxicillin	635 (17.8)	22 (0.6)	1673 (47.0)	932 (26.2)	297 (8.4)	3559 (27.7)
J01FA10	Macrolides/Azithromycin	77 (5.3)	29 (1.9)	300 (20.5)	697 (47.7)	359 (24.6)	1462 (11.4)
J01AA02	Tetracyclin/Doxycyclin	21 (2.1)	8 (0.8)	212 (21.2)	724 (72.4)	35 (3.5)	1000 (7.8)
J01CA08	Pivmecillinam	0 (0.0)	953 (99.8)	1 (0.1)	1 (0.1)	0 (0.0)	955 (7.4)
J01DB01	Cephalosporin/Cephalexin	398 (65.6)	11 (1.8)	77 (12.7)	79 (13)	42 (6.9)	607 (4.7)
J01EE01	Sulfamethoxazole and trimethoprim	65 (18.3)	267 (75.2)	0 (0.0)	23 (6.5)	0 (0.0)	355 (2.7)
J01XE01	Nitrofurantoin	0 (0.0)	301 (99.7)	1 (0.3)	0 (0.0)	0 (0.0)	302 (2.4)
J01EA01	Trimethoprim	3 (1.0)	292 (99.0)	0 (0.0)	0 (0.0)	0 (0.0)	295 (2.3)
J01MA02	Fluorocinolones/ Ciprofloxacin	1 (0.4)	242 (96.0)	4 (1.6)	5 (2.0)	0 (0.0)	252 (2.0)
J01CE02	Phenoxymethylpenicillin	122 (52.0)	0 (0.0)	91 (38.7)	13 (5.5)	9 (3.8)	235 (1.8)
J01FA01	Macrolides/Erythromycin	7 (11.1)	0 (0.0)	27 (42.9)	16 (25.4)	13 (20.6)	63 (0.5)
J01DC02	2. Generations cephalosporines	6 (26.1)	1 (4.4)	6 (26.1)	5 (21.7)	5 (21.7)	23 (0.2)
J01FA09	Macrolides/Clarithromycin	4 (28.6)	0 (0.0)	4 (28.6)	3 (21.4)	3 (21.4)	14 (0.1)
J01CF01	Dicloxacillin	6 (50.0)	3 (25.0)	1 (8.3)	1 (8.3)	1 (8.3)	12 (0.1)
J01FF01	Lincosamides/Clindamycin	1 (14.3)	1 (14.3)	3 (42.9)	0 (0.0)	2 (28.5)	7 (0.1)
J01XX05	Methenamin/Haiprex	0 (0.0)	1 (100)	0 (0.0)	0 (0.0)	0 (0.0)	1 (0.0)
		2707	2144	3515	3206	1278	12,850

*Percentage in rows shows antibiotic prescribed per diagnosis. The end column shows percentage of total antibiotic prescriptions.

The most common antibiotic prescribed in total was amoxicillin with clavulanic acid, 5695 prescriptions (22.7%). The second most prescribed antibiotic in total was amoxicillin, 4608 prescriptions (18.4%). The third most common antibiotic prescribed in total was phenoxymethypenicillin, total of 4227 prescriptions (16.9%). Table 1 reveals the number (%) of all antibiotic prescriptions for the five diagnosis selected in the study. Table 2 reveals numbers and distribution of antibiotics prescribed by diagnosis and age group and the proportion of antibiotic prescriptions for each diagnosis within age group.

**Table 2.** Numbers and distribution of antibiotics prescribed by diagnosis and age group (% within age group).

	Age group 0–4 y	Age group 5–17 y	Age group 18–60 y	Age group >60 y	Total antibiotics prescribed
Bronchitis	164 (7.5)	198 (13.7)	2060 (27.6)	784 (44.9)	3206
Cystitis	59 (2.7)	232 (16.1)	1481 (19.8)	372 (21.3)	2144
Otitis media	1704 (77.8)	500 (34.7)	469 (6.3)	34 (1.9)	2707
Pneumonia	201 (9.2)	217 (15.0)	607 (8.1)	253 (14.5)	1278
Sinusitis	61 (2.8)	295 (20.5)	2855 (38.2)	304 (17.4)	3515
Total antibiotics prescribed n	2189	1442	7472	1747	12,850

**Figure 1.**
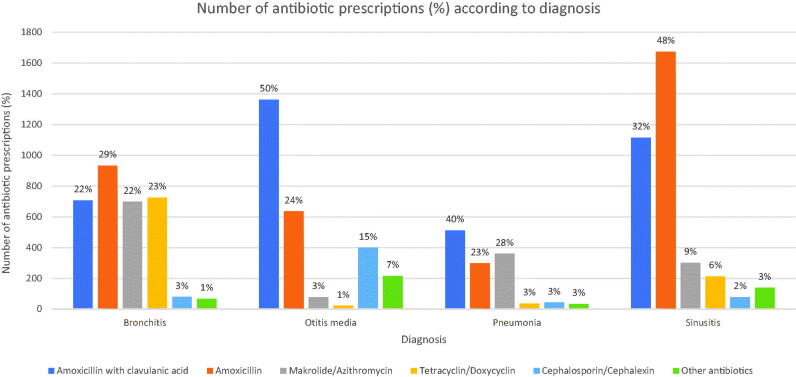
Number of antibiotic prescriptions (%) according to diagnosis.

Figure 1 shows the distribution of antibiotics prescribed in total for each diagnosis. Amoxicillin with clavulanic acid was proportionally most often prescribed in OM or in 50% of all OM diagnosis, secondly for pneumonia or 40% of diagnosis.

Table 3 shows the number of antibiotics prescribed for cystitis, divided to age groups and proportion of antibiotics prescribed within age group. The difference in choice of an antibiotic for different age groups is likely to be explained by the form of the antibiotic, as sulfamethoxazole and trimethoprim/sulfamethoxazole are found in suspension. Table 4 shows the number of antibiotics prescribed for bronchitis, otitis media, pneumonia and sinusitis diagnosis, divided to age group, and proportion of antibiotic prescribed within each age group.

**Table 3.** Numbers of antibiotics prescribed for cystitis divided to age groups (% within age group).

	Amoxicillin and clavulanic acid	Amoxicillin	Piv-mecillinam	Sulfamethoxazole and trimetho-prim	Nitrofuran-toin	Trimetho-prim	Fluorocin-olones/ Ciprofloxacin	Other antibiotics
**Cystitis**	13	22	953	267	301	292	242	54
0–4y	7 (11.9)	3 (5.1)	1 (1.7)	25 (42.4)	5 (8.5)	17 (28.8)	0 (0.0)	1 (1.6)
5–17y	2 (0.9)	3 (1.3)	79 (34.1)	52 (22.4)	33 (14.2)	50 (21.6)	5 (2.2)	8 (3.3)
18–60y	3 (0.2)	12 (0.8)	731 (49.4)	153 (10.3)	197 (13.3)	190 (12.8)	153 (10.3)	42 (2.9)
>60y	1 (0.3)	4 (1.1)	142 (38.2)	37 (9.9)	66 (17.7)	35 (9.4)	84 (22.6)	3 (0.8)

**Table 4.** Number of antibiotics prescribed per diagnosis and age group (percent within age group).

	Amoxicillin and clavulanic acid	Amoxicillin	Macrolides/ Azithromycin	Tetracyclin/ Doxycyclin	Cephalosporine/ Cephalexin	Other antibiotics
Bronchitis	707	932	697	724	79	67
0–4y	76 (46.3)	17 (10.4)	23 (14.0)	0 (0.0)	30 (18.3)	18 (11.0)
5–17y	48 (24.2)	72 (36.4)	58 (29.3)	10 (5.1)	3 (1.5)	7 (3.5)
18–60y	405 (19.7)	642 (31.1)	473 (23.0)	483 (23.4)	24 (1.2)	33 (1.6)
>60y	178 (22.7)	201 (25.6)	143 (18.2)	231 (29.4)	22 (2.8)	9 (1.3)
Otitis media	1361	635	77	21	398	215
0–4y	973 (57.1)	220 (12.9)	39 (2.3)	0 (0.0)	356 (20.9)	116 (6.8)
5–17y	189 (37.8)	218 (43.6)	10 (2.0)	3 (0.6)	27 (5.4)	53 (10.6)
18–60y	179 (38.2)	187 (39.9)	27 (5.8)	16 (3.4)	15 (3.2)	45 (9.5)
>60y	20 (58.8)	10 (29.4)	1 (2.9)	2 (5.9)	0 (0.0)	1 (3.0)
Pneumonia	512	297	359	35	42	33
0–4y	117 (58.2)	24 (11.9)	24 (11.9)	0 (0.0)	31 (15.4)	5 (2.6)
5–17y	68 (31.3)	57 (26.3)	80 (36.9)	3 (1.4)	1 (0.5)	8 (3.6)
18–60y	219 (36.1)	147 (24.2)	197 (32.5)	26 (4.3)	4 (0.7)	14 (2.2)
>60y	108 (42.7)	69 (27.3)	58 (22.9)	6 (2.4)	6 (2.4)	6 (2.4)
Sinusitis	1115	1673	300	212	77	138
0–4y	39 (63.9)	5 (8.2)	1 (1.6)	0 (0.0)	11 (18.0)	5 (8.3)
5–17y	88 (29.8)	155 (52.5)	20 (6.8)	11 (3.7)	7 (2.4)	14 (4.8)
18–60y	889 (31.1)	1391 (48.7)	246 (8.6)	172 (6.0)	48 (1.7)	109 (3.9)
>60y	99 (32.6)	122 (40.1)	33 (10.9)	29 (9.5)	11 (3.6)	10 (3.3)

## Discussion

This is, to our best knowledge, the first study on antibiotic prescriptions in OOH service in Reykjavik capital area. The main findings are that antibiotics were prescribed in one out of three consultations and almost 30% were prescribed amoxicillin with clavulanic acid. Studies from Denmark, Norway and England have revealed an overall antibiotic prescription rate in OOH service around 15% [6,13,15]. Other diagnoses antibiotics were prescribed for in the study period, apart from the diagnoses we selected in the study, were mainly other respiratory tract infections (including tonsillitis) (20.3%), skin infections (11.2%) and urinary tract infections and gastrointestinal infections (2.0%). The most commonly prescribed antibiotic in OOH service in Scandinavian countries is phenoxymethylpenicillin, or up to 70% of all antibiotic prescriptions [6,7,13,14]. Amoxicillin, was most often prescribed in OOH service in England or in 28.2% of total antibiotic prescriptions [5]. A study on antibiotic prescriptions during daytime hours and OOH in The Netherlands showed, that amoxicillin with clavulanic acid was most often prescribed in OOH service, 22% of antibiotic prescriptions, and secondly amoxicillin, 21% of antibiotic prescriptions in OOH service [11]. In the Netherlands, as in Iceland, amoxicillin is the first-line recommended antibiotic for acute otitis media (AOM). Debets et al. found that 85.9% of diagnosed AOM in OOH service in The Netherlands, were prescribed amoxicillin, according to clinical guidelines [11]. We found that 50.3% of all AOM diagnosed, were prescribed a more broad-spectrum antibiotic, amoxicillin with clavulanic acid, and only 23.5% of all OM were prescribed amoxicillin, according to clinical guidelines in Iceland (Figure 1). When studies are evaluated with regards to prescriptions of antibiotics according to guidelines, a study from OOH service in Norway revealed that 69% of antibiotic prescriptions were according to guidelines [6]. The proportion of antibiotic prescriptions according to guidelines in a Dutch study, which was for both OOH and daytime hours revealed the same, 69% [11]. A Danish study on antibiotic prescriptions in all general practice, showed that 58% of antibiotic precriptions was penicillin V for ARTIs [14]. A study from Belgium, where quality of antibiotic prescription during daytime hours and OOH primary care was studied using European quality indicators, discovered that cystitis was the only condition in which the percentage of patients prescribed an antibiotic was within the proposed range of acceptable use [8]. The same study showed that less than half of the prescribed antibiotics were the recommended ones [8].

We do not have comparative studies on antibiotic prescriptions for daytime services in primary care in Iceland, but studies from other countries have tried to illustrate the difference in antibiotic prescription rate for OOH and daytime hours in primary care [5,7,8,11,14]. One might suggest that patients who seek OOH are more ill and they find that they cannot wait until the next day to see their GP [5,6,11,13,16]. A study from Belgium, where quality of antibiotic prescription during daytime hours and OOH primary care was studied, revealed that a similar patient population and illness severity is presenting during daytime hours and OOH [8]. A study from England demonstrated trend in prescribing that could represent a partial displacement of antibiotic prescribing from daytime hours to OOH, due to patients antibiotic-seeking behaviour after refusal by the daytime hours GP [5]. Studies have also found that factors particular to influence antibiotic prescription in OOH service, include lack of patient follow-up, lack of access to patient GP records and short consultation time [5,7,13,16]. In a study from the UK, GP´s recorded how consultation time and the pressure to end a consultation influenced the likelihood of prescribing an antibiotic during a busy OOH shift [16]. A GP under time pressure, may feel that it is time consuming to discuss alternative approaches with the patient, and therefore chooses to prescripe antibiotics [5–7,16]. A Norwegian study indicated that GP´s with a high number of total annual encounters had higher antibiotic prescription rates for ARTIs and used more non-first line antibiotics compared to GP´s with fewer annual patient encounters [19].

In OOH primary care service in Reykjavik capital area there are no nurses that triage the consultations as in OOH service in some other countries. Therefore, people can go directly to see a GP and no one is denied access. People may find it convenient to avoid abstaining from work, as they can see a GP directly in OOH service, and this has also been suggested in other Scandinavian studies [6,13]. In this study, we did not take a look at the GP´s notes on the consultation, therefore we were not able to differentiate whether antibiotic prescriptions occured after initial treatment failure which can be a factor in high rate of broad-spectrum antibiotic prescriptions. In Iceland, as in Norway, higher number of GP´s OOH´s sessions generates higher income and also higher number of consultations per session, generates higher income (up to a limit amount) [6]. This may trigger an economic incentive for some GP´s. A possible part of the explanation of broad-spectrum antibiotic prescription of amoxicillin with clavulanic acid for the youngest age group particularly, is that amoxicillin suspension was not possible to prescribe electronically in Iceland between august 2013 and august 2015. Physicians could still prescribe the amoxicillin suspension, but it required a written form, which was more time consuming. An Icelandic study on the impact of the 10-valent pneumococcal conjugate vaccine PHiD-CV on antimicrobial prescriptions in young children, discovered that 18 months after the amoxicillin suspension was again available in electronic prescription form, its use was still lower than before it was presented in written form [20]. The same study revealed that the introduction of PHiD-CV10 vaccine in Icelandic vaccination program in 2011, lead to reduced antimicrobial use in children, mainly by reducing acute otitis media episodes [20].

### Strengths and limitations

This study is a retrospective data analysis. The main strength of this study is that there is one place for primary care OOH service for Reykjavik capital area with electronic data. The results reflect the current reality as registered in the electronic patient records. The GP´s involved were not aware of the plans of a research at the time of the consultations. Antibiotic prescriptions were not studied specifically for all possible diagnosis or indications for antibiotic treatment. We only studied explicitly common, rather well-defined diagnosis. Since the result showed that these diagnosis only accounted for 50% of all prescriptions, it is a certain limitation.

This study is based on the diagnosis given at the consultation, which is a limitation, as there may be a lack of accuracy in making diagnosis. We have no possibility to differentiate between those who were at the OOH service due to first-line antibiotic treatment failure, follow up due to former started treatment or amongst those who had been told to wait and see. The assesment of guidelines often requires facts that are recorded in plain text, such as history, clinical signs, severity of infections, whether first-line antibiotic treatment has failed and utilisation of rapid tests.

### Implications

These results can give the OOH service in primary care an opportunity to reassess the prescriptions of antibiotics. Increased awareness of antibiotic resistance and guidance of reducing antibiotic prescriptions are among important steps neccessary to take. Further studies would benefit from differentiating between daytime hours and OOH antibiotic prescriptions in the capital area of Reykjavík, and whether quantity and quality of antibiotic prescription has improved in OOH service since 2014. Furthermore, clinical guidelines, reflecting the respective bacterial resistance, are required at least for the most common ARTIs.

## Conclusion

This study shows a high rate of antibiotic prescription´s in OOH primary care service in Reykjavik capital area and a high prescription rate of broad-spectrum antibiotics. Results that indicate an opportunity to improve the quality and to develop a more rational prescription of antibiotics in primary care in Iceland.
